# Current State of the Science: Health Effects and Indoor Environmental Quality

**DOI:** 10.1289/ehp.8987

**Published:** 2007-01-25

**Authors:** Clifford S. Mitchell, Junfeng (Jim) Zhang, Torben Sigsgaard, Matti Jantunen, Paul J. Lioy, Robert Samson, Meryl H. Karol

**Affiliations:** 1 Department of Environmental Health Sciences, Johns Hopkins Bloomberg School of Public Health, Baltimore, Maryland, USA; 2 Department of Environmental and Occupational Health, School of Public Health, University of Medicine and Denistry of New Jersey, Piscataway, New Jersey, USA; 3 Department of Environmental and Occupational Medicine, University of Aarhus, Aarhus, Denmark; 4 Department of Environmental Health, National Public Health Institute of Finland, Kuopio, Finland; 5 Department of Environmental and Community Medicine, Robert Wood Johnson Medical School, University of Medicine and Denistry of New Jersey, Piscataway, New Jersey, USA; 6 Department of Services and Applied Research, Centraalbureau voor Schimmelcultures, Utrecht, the Netherlands; 7 Department of Environmental and Occupational Health, School of Public Health, University of Pittsburgh, Pittsburgh, Pennsylvania, USA

**Keywords:** allergens, chemistry, exposure, fungi, humans, indoor air pollution, intervention, review

## Abstract

Our understanding of the relationship between human health and the indoor environment continues to evolve. Previous research on health and indoor environments has tended to concentrate on discrete pollutant sources and exposures and on specific disease processes. Recently, efforts have been made to characterize more fully the complex interactions between the health of occupants and the interior spaces they inhabit. In this article we review recent advances in source characterization, exposure assessment, health effects associated with indoor exposures, and intervention research related to indoor environments. Advances in source characterization include a better understanding of how chemicals are transported and processed within spaces and the role that other factors such as lighting and building design may play in determining health. Efforts are under way to improve our ability to measure exposures, but this remains a challenge, particularly for biological agents. Researchers are also examining the effects of multiple exposures as well as the effects of exposures on vulnerable populations such as children and the elderly. In addition, a number of investigators are also studying the effects of modifying building design, materials, and operations on occupant health. Identification of research priorities should include input from building designers, operators, and the public health community.

Our understanding of health effects related to the indoor environment has evolved over the past decade. In the past, discussions of indoor environmental quality (IEQ) focused on indoor air constituents (primarily particles, bioaerosols, and chemicals), and comfort factors (temperature, air flow, and humidity) (Samet et al. 1998). More recently, we have begun to look at the relationship between the built environment and humans as a complex interplay between building occupants (who they are and what they do) and an array of physical, chemical, biological, and design factors. This evolution in understanding has profound implications for the design and operation of buildings, how the buildings are used, and the prevention and management of health problems that occur in building occupants.

## Source Characterization

Outdoor air pollution is a dynamic system in which the physical and chemical processes affecting the accumulation of pollutants in the atmosphere are constantly changing, largely driven by complex meteorology and photochemistry. In contrast, the usual approach of modeling indoor air pollution considers only pollution source strength and dilution by air exchange, thus treating the indoor environment as a static box in which physical and chemical transformations of indoor air pollutants are absent or negligible. This misconception produces conservative estimates for primary indoor air pollutant concentrations and ignores the secondary pollutants. In-depth studies of indoor air have shown that the concentration of agents in indoor air is a function of outdoor concentration, indoor source strength, removal and deposition rate within the structure, indoor mixing, and chemical reaction. In the following sections, we use real-world examples to illustrate the dynamic nature of these processes and to discuss the implication of this dynamic environment in assessing exposures and health effects associated with indoor air pollution.

### Indoor production

The generation of pollutants within the indoor environment may come from primary and secondary sources. Primary sources include fuel combustion for cooking, heating, and lighting; tobacco smoking; bioeffluents from humans and animals; floor and wall coverings; synthetic paints, glues, polishes, and waxes; pesticides; and building products. Another source is the release of gases from solvents used indoors or from water that is used daily for showers, bathing, cooking, and from drinking fountains. Such sources are important for by-products (e.g., chloroform) of chlorination-based water disinfection and radon (McKone and Knezovich 1991; Xu and Weisel 2005). Because of the use of many types of synthetic materials in our daily lives, concentrations of many volatile organic compounds (VOCs) are consistently higher indoors than outdoors in residences and offices in developed countries. For some VOCs such as limonene, indoor levels up to 10 times those outdoors are common, even in locations with significant outdoor air pollution sources, such as petrochemical plants (Ott and Roberts 1998; Weisel et al. 2005). Secondary sources refer to indoor chemistry that transforms a set of indoor pollutants, emitted from primary sources or transported from outdoors, to a new set of indoor pollutants, as discussed below.

### Outdoor-to-indoor transport

Pollutants of outdoor origin, including those present in the outdoor air and those released from soil sources, can be transported indoors via building openings and cracks (Garbesi et al. 1999; Nazaroff 2004). Attempts have been made to estimate the fraction of measured indoor concentration contributed by outdoor air due to the outdoor-to-indoor transport process (Ott et al. 2000; Thatcher and Layton 1995). One such study, the Exposures of Adult Urban Populations in Europe Study (EXPOLIS), compared concentrations of ambient particulate matter ≤ 2.5 μm (PM_2.5_), its 16 elemental constituents and black carbon, 30 VOCs, and carbon monoxide (CO) among urban adult populations in seven European cities. The study examined exposures in workplaces, residential outdoor and indoor air, and separated workday and leisure time (Jantunen et al. 1998). EXPOLIS data from Helsinki, Finland, showed the infiltration factor (the proportion of outdoor PM found indoors) for PM_2.5_ averaged 0.64 for residential structures, 0.47 for workplaces, and 0.35 for a subsample of office buildings constructed after 1990 (Hänninen et al. 2004b, 2005). In another study, the Relationship of Outdoor, Indoor, and Person Air (RIOPA), fractions of measured indoor concentration contributed by outdoor air for PM_2.5_ and each of 24 VOCs including 10 aldehydes and ketones were estimated for 310 residences located in three U.S. cities (Weisel et al. 2005). The median fractions of measured indoor concentration contributed by outdoor air for compounds with dominant indoor sources were less than 50%, for example, 13% for *d*-limonene (a common cleaning solvent), 20% for chloroform (a byproduct of drinking water disinfection), 31% for α-pinene and 20% for β-pinene (ingredients of synthetic paints), and 19% for formaldehyde (released from building/furnishing materials). For the compounds with sole or dominant outdoor sources (e.g., methyl *tert* butyl ether, carbon tetrachloride, and trichloroethylene), the fractions were about 100%, as expected. The fractions for PM_2.5_ had a median of 56%, 25th percentile of 46%, and 75th percentile of 93% across the RIOPA homes (Meng et al. 2005; Weisel et al. 2005).

Significant interhome variability in fractions of measured indoor concentrations contributed by outdoor air has been observed for PM_2.5_ and most of the VOCs in the RIOPA study. This finding has important implications for air pollution epidemiologic studies using concentrations measured at outdoor locations. Numerous exposure studies have shown poor correlations between personal exposure or residential indoor concentration and outdoor concentrations, indicating the observed associations between adverse health effects and PM concentrations measured at fixed outdoor sites do not necessarily represent the exposure–response relationships (Adgate et al. 2004; Clayton et al. 1993). Although attempts have been made to differentiate PM of outdoor origin from PM of indoor origin, analyses have been complicated because the fraction of indoor species contributed by outdoor air depends not only on outdoor concentration but also on home-specific parameters including air exchange rate [AER; typically expressed as air exchanges per hour (ach)], indoor generation rate, removal rate, and house volume (Meng et al. 2005; Thomas et al. 1993; Wallace et al. 1991).

Outdoor-to-indoor transport of very reactive chemical species has often been considered unimportant. An example is ground-level ozone (O_3_) that is formed via photochemical reactions and has elevated concentration in polluted atmospheres during photochemical smog episodes. O_3_, like PM, is regulated in the United States as a criteria pollutant. Because of its high reactivity, only a fraction of O_3_ can penetrate a building envelope. This fraction had been considered insignificant to cause any exposure concerns until 1989 when Weschler et al. (1989) showed that indoor exposure to O_3_ can easily surpass outdoor exposure. Under moderate AERs (~ 0.5 ach), indoor O_3_ concentrations may be 20–30% of corresponding outdoor concentrations. Under high AERs (> 1 ach), indoor O_3_ levels can be 50–70% of outdoor levels. In a study carried out in six homes located in suburban New Jersey, indoor O_3_ concentrations were 22–66% of outdoor levels during afternoon hours (Zhang et al. 1994). In summer time, 50% of the schools measured in Mexico City had indoor O_3_ levels > 113 ppb (Gold et al. 1996). It is reasonably conservative to state that indoor O_3_ levels > 20 ppb are common when outdoor O_3_ concentrations are elevated. O_3_ concentration at 20 ppb may not be sufficient to cause health concerns due to direct O_3_ exposure, but this O_3_ level can be sufficient to drive a complex set of indoor chemical reactions. When O_3_ generators (so-called air purifiers) are used at O_3_ generation rates of tens to thousands of milligrams per hour, indoor O_3_ concentrations can be in the parts per million levels in a room with typical volume and AER.

Particle sources include both indoor home and residential sources, although recent research has shown that indoor (workplace and residential) contributions to total exposures may be underestimated compared with outdoor sources such as traffic (BeruBe et al. 2004; Koistinen et al. 2004). This appears to depend on the character of the particle; combustion-derived particles may be due more to outdoor sources, whereas other particles (for example, soil-derived particles) may be related to resuspension of particles during a host of indoor activities (Ferro et al. 2004; Larson et al. 2004). Recent experiments have shown that a wide range of indoor activities can result in considerable generation of PM (Afshari et al. 2005). Models of indoor PM exposure have been developed to account for both indoor and outdoor sources, as well as mixing, transport, and removal (Georgopoulos et al. 2005; Nazaroff 2004).

### Indoor-to-outdoor transport

Ventilation is the primary factor affecting indoor-to-outdoor transport of indoor generated pollutants. Ventilation is necessary to reduce concentrations of pollutants generated indoors, but it is also necessary to reduce the time available for chemical reactions among indoor pollutants. One reason offered to support the conventional view of indoor chemistry being insignificant is that chemical reactions among indoor pollutants are too slow to complete with air exchange processes. Although this may be true when the AER is high, a variety of chemical reactions can take place at AERs typical of today’s residences and offices. Since the late 1970s, the airtight design of buildings, driven mainly by energy conservation, has resulted in reduced AERs. Based on approximately 4,590 measurements of residential AERs conducted across the United States, Pandian et al. (1998) reported that the mean, median, and SDs of AERs were 0.55, 0.42, and 0.47 ach, respectively, for the northeastern region, and 0.71, 0.62, and 0.56 ach for the southeastern region of the United States. AERs of this magnitude are undesirable for removing air pollutants that originate indoors and are low enough for certain chemical reactions to occur.

### Indoor chemistry

Pollutants can be removed from indoor air through both physical and chemical processes. Physical processes that can result in pollutant removal (in addition to transport outdoors) include phase change, adsorption or absorption, or dissolving in water or organic films. Recently there has been considerable research interest in removal of pollutants through chemical reactions.

“Indoor chemistry” has been defined as reactions involving indoor pollutants, occurring either in the gas phase or on surfaces (Weschler et al. 2006). For a chemical reaction to influence the indoor environment, the rate of the reaction must be sufficient to compete with AERs. These chemical reaction processes represent sinks for the reactants (primary indoor pollutants) and sources of new reaction products (secondary indoor pollutants). The products may predominate in the air or on the surface. Removal does not necessarily occur in a simple linear fashion; for example, semi-volatile organic compounds can undergo an initial removal followed by a secondary increase due to resuspension of the compounds adsorbed on particles (Lioy 2006).

Both gas-phase reactions and surface reactions that can occur under typical indoor conditions have been identified. The most extensively studied gas-phase reactions are oxidation reactions involving O_3_ and free radicals. O_3_ drives most indoor oxidation chemistry because it can react at meaningful rates with nitric oxide, nitrogen dioxide, and unsaturated organic compounds (e.g., terpenes, terpenoids, sesquiterpenes, unsaturated fatty acids) to yield reactive intermediates, the hydroxyl radical (OH), the nitrate radical (NO_3_) and oxygenated organic compounds (Weschler and Shields 1996). Reactions of O_3_ with NO_2_, in the absence of sunlight, form the NO_3_ radical that further reacts with VOCs, leading to the formation of indoor nitric acid. The NO_3_ radical can also react with NO_2_ to form dinitrogen pentaoxide (N_2_O_5_) that undergoes hydrolysis, another pathway of nitric acid formation (Weschler et al. 1992). When O_3_ and NO_2_ are present simultaneously, indoor NO_3_ may be the dominant indoor oxidant that effectively reacts with nearly all indoor VOCs. The role of indoor NO_3_ chemistry in transforming indoor air pollutants remains to be evaluated.

Several terpenes, especially *d-*limonene and α-pinene, are present at substantially higher concentrations indoors compared those with outdoors. These terpenes react readily with O_3_ under typical or realistic indoor conditions to initiate a series of complex chemical reactions, for example, at an O_3_ concentration of 20 ppb, the rate constant for O_3_ reaction with *d*-limonene and α-pinene is approximately 0.36 ach and approximately 0.15 ach (Fan et al. 2003). Products of these reactions are found in both the gas and particle phases. Gas-phase–stable products include aldehydes, carboxylic acids, potentially allergenic peroxides and hydroperoxides (Fan et al. 2003). In one experiment where O_3_ (~ 41 ppb) was mixed with a VOC mixture comprising 23 commonly found VOCs, the resulting peak concentration of ultrafine and fine particles was approximately 100 μg/m^3^ (Fan et al. 2005). Although attempts have been made to chemically identify the resulting particles, the majority of the particle mass could not be explained by the compounds identified thus far (Fan et al. 2003). It will be even more challenging to identify the short-lived, highly reactive, thermally labile or highly oxidized species that are formed in this complex reaction system. Unstable products of the ozone–terpene reactions include reactive intermediates and the hydroxyl radical. Hydroxyl radicals resulting from these indoor reactions can reach levels higher than typical nighttime outdoor concentrations, and thus react with other indoor VOCs with which ozone reacts too slowly to be of any practical significance (Weschler and Shields 1996).

The formation of particles via O_3_-driven indoor chemistry has two implications. First, in an analysis of indoor particles measured in residences located in several United States cities, 25% of indoor PM_2.5_ could not be explained with known sources (Wallace 1996). Indoor chemistry was not considered in the analysis, which might explain at least part of the unknown sources. Second, because O_3_ and fine particles are co-generated outdoors during photochemical episodes, indoor particles resulting from indoor O_3_/VOC reactions can vary coincidently with the variations of outdoor summertime fine particles. This will certainly complicate the effort to separate PM of outdoor origin from PM of indoor origin. It should also be noted that source characterization may vary significantly, depending on the size of the particles (Koistinen et al. 2004).

A second type of indoor chemistry involves surface reactions. Outdoor aerosol surfaces play an important role in atmospheric chemistry. The importance of surface reactions indoors is easily recognized, given that surface-to-volume ratios indoors are much larger than outdoors (roughly 3 vs. 0.01 m^2^/m^3^). Indeed, indoor surfaces may be ideal for substance sorption and for water condensation. Surface water film can react with indoor NO_2_, a major product of natural gas combustion, to form nitrous acid (HONO) and nitric acid (HNO_3_). The resulting nitrous acid is released into the air as gas-phase HONO, whereas nitric acid remains on surfaces as an HNO_3_–H_2_O complex (Dubowski et al. 2004). The latter yields possible acidic, oxidizing, and nitrating surface films on interior walls. O_3_ reacts with unsaturated VOCs contained in surface coatings at a faster rate than when it reacts with the same compounds in the gas phase (Reiss et al. 1995).

Indoor surfaces, including building materials, wall cavities, ducts, skin, clothing, dust, and airborne particles are very diverse and are a determining factor of indoor surface chemistry. They affect HONO formation via surface-NO_2_ chemistry (Wainman et al. 2001). Complex physical and chemical processes involving surfaces include sorption, redox reactions, acid-base chemistry and hydrolysis (Nazaroff and Singer 2004). For example, diphthalate esters (plasticizers contained in polyvinyl chloride flooring materials) can undergo hydrolysis to form alcohols and monoesters. Aldehydes are emitted, at concentrations exceeding their odor thresholds, when O_3_ interacts with carpets (Morrison and Nazaroff 2002).

Building materials contain a large number of reactive constituents that can be released into the indoor air along with secondary products, including terpenoids, aliphatic aldehydes, phthalates, phenol, mono- and dicarboxylic acids, diisocyanates, and various photoinitiators. Photoinitiators, contained in ultraviolet curable coatings, can undergo decomposition to generate free radicals, and some (e.g., benzaldehyde and cyclohexanone) are precursors of odorous products (Salthammer et al. 2002). In a study conducted in German houses constructed with wooden studs treated with pentachlorophenol (PCP), it was found that over time PCP had been transformed to tetra-chloroanisole, a compound of highly undesirable odor (Gunschera et al. 2004).

Indoor oxidation chemistry is largely driven by O_3_ reactions with unsaturated VOCs and perhaps with NO_2_ as well. Given that ozone levels have been rising in many areas, that indoor use of unsaturated VOCs (e.g., terpenes) has been on the rise, and that AERs have been decreasing, indoor oxidation chemistry has likely increased over the past several decades.

## Exposure Assessment

Much remains to be learned about exposure assessment in indoor environments. Part of the challenge is to account for the relative contributions of both indoor and outdoor exposures. This has important implications, as indoor and outdoor exposures are often regulated very differently. Studies suggest that although indoor environmental measurements provide a better estimate of personal exposure than outdoor monitoring of VOCs, neither indoor nor outdoor environmental sampling (together or individually) is a good predictor of personal exposures (assessed by personal sampling and blood VOC concentrations) (Sexton et al. 2004, 2005).

Exposure assessment for biological agents is even more challenging than for particulate and chemical exposures. New and more accurate identification methods to identify molds are under development. Currently, polymerase chain reaction (PCR) methods are used in which the target DNA from building material is used as a template. In quantitative PCR (qPCR), quantitative data on the presence of viable and dead molds can be obtained—information that is not possible to obtain with the present culture methods (Cruz and Stetzenbach 2004; Meklin et al. 2004). These new methods are not yet fully developed and need to be evaluated (Keswani et al. 2005; McDevitt et al. 2004; Vesper et al. 2004). Even if fungal and mold species can be identified more accurately in the environment, there are as yet no reliable markers of human exposure or dose for these and other biological agents; some efforts are under way to assess exposure using chemical markers or immunologic markers (Schmechel 2006; Sebastian et al. 2005).

## Health Effects

In this section we review recent findings on specific agents and mixtures of pollutants. Some of the most significant advances have been made in our understanding of the mechanism of inflammation, and its role in mediating the responses to a wide variety of environmental stressors.

### Particulate matter

Particulate air pollution has long been linked to both acute and chronic health effects, including asthma (e.g., mineral and organic dusts), cardiac disease (e.g, tobacco smoke and ambient air PM_2.5_), and other conditions (Pope et al. 1991; Viegi et al. 2004). Recent attention has focused on the ability of PM to potentiate the effects of common allergens, promoting IgE production (Karol 2002). Fine particles have been shown to decrease the forced expiratory volume in 1 sec (FEV_1_) in asthmatic schoolchildren (Delfino et al. 2004). Although particles have been shown to increase cardiovascular mortality, the specific mechanisms by which this occurs have yet to be clarified. Recent investigations have focused on possible effects on heart rate variability (Magari et al. 2002; Pope et al. 1999). PM, especially products of combustion, has also been linked to the development of cancer, although the exact relationship is still under active investigation (Vineis and Husgafvel-Pursiainen 2005).

Most studies of PM have focused on ambient (outdoor) exposures and their relationship to hospital admissions and mortality. The contribution and significance of indoor particulate matter, which may differ substantially in composition from outdoor particulates, have yet to be fully explored (Bell et al. 2004; Morris 2001). Few studies have described the attributable risk of adverse health effects from indoor sources of particles, but some are attempting to quantify the relative contributions of indoor and outdoor particulate matter (and other toxins) in greater detail, to aid risk and exposure models (Weisel et al. 2005).

### Chemicals

Chemicals of interest in the built environment include volatile and semi-volatile organic compounds, pesticides, and some chemicals produced during combustion (carbon monoxide, nitrogen oxides). Initially, interest in chemicals in indoor environments focused primarily on irritant and toxic properties of individual chemicals such as volatile organic compounds (VOCs) and combustion products. Concerns were also raised about the potential for chronic health effects (primarily cancer) related to exposures to organic compounds. There is interest also in the health effects from plastics and plasticizers. Chemical constituents of plastics have been found in household dust, and studies suggest these plasticizers may be related to allergic diseases in children (Bornehag et al. 2004b, 2005; Oie et al. 1997). Chemical processing inside structures also contributes to adverse health effects from indoor chemicals (Weschler 2004).

The relationship between irritation, stress, and perceived health effects of VOC exposures has gained increased attention. In one recent study, controlled exposures to VOCs, with and without ozone, did not significantly affect health effects compared with performance of a stress-inducing task (Fiedler et al. 2005).

The relationship of VOCs to asthma, particularly in children, remains controversial. A population-based case–control study of asthmatic and nonasthmatic children (ages 6 months to 3 years) in Australia found that the adjusted odds ratios for asthma increased with increasing concentrations of VOCs (particularly benzene, toluene, ethylbenzene, and xylene) (Rumchev et al. 2004). By contrast, a study in the United Kingdom found that VOC exposure (except formaldehyde) was not associated with an increased risk of wheezing illness, whereas dampness was significantly associated with wheezing illness (Venn et al. 2003). Several factors could account for inconsistencies between observational and interventional studies of home exposures to VOCs and asthma risk, including confounding, small effect levels, or chronicity of exposure (Dales and Raizenne 2004).

Polybrominated diphenyl ethers commonly used in flame retardants in consumer products can concentrate in house dust, and thus are potentially available for ingestion by occupants (Gevao et al. 2006). Similar results have been obtained for a variety of chemicals used in consumer products, indicating the importance of examining not only building components but also furnishings and contents of the indoor environment as sources of exposure (Marklund et al. 2003).

### Biological agents

#### Animal antigens

Allergy to indoor agents can cause frequent and severe health problems, especially in children. Animal allergens are found commonly indoors, even where animals are not present. For example, assessment of cat, dog, and mite allergens in settled dust in schools and day-care centers in Oslo, Norway, revealed most samples contained detectable amounts of cat and dog allergens. Allergens were detected in mattress and floor dust in daycare centers and in curtain and floor dust in schools. The levels of cat and dog allergens in school floor dust were associated with the number of pupils with animals at home. By contrast, < 1% of the samples had measurable levels of mite allergen Der p 1. Endotoxin levels were also assessed. Levels of endotoxin tended to be higher in dust from floors (1.4 ng/m^2^) compared with that from mattresses (0.9 ng/m^2^). Mattresses in daycare centers are reservoirs of cat and dog allergens and should be cleaned frequently (Instanes et al. 2005).

In most communities, avoiding cats in the home would not decrease the prevalence of sensitization to cats because cat allergen is distributed in schools, other public buildings, and homes without a cat. Evidence that children or adults who make a modified T-helper 2 response (IgG and IgG4 antibody without IgE) are not at increased risk of asthma supports the role of IgE in asthma (Erwin et al. 2005).

#### Biological hazards associated with damp indoor environments

There is a large and growing literature on the health effects of biological agents typically found in damp indoor environments (Bornehag et al. 2001, 2004a). An Institute of Medicine (IOM) committee concluded there was sufficient evidence of association of damp indoor spaces with various upper and lower respiratory tract symptoms in adults and children. Molds and other specific biological agents were associated with a number of conditions including hypersensitivity pneumonitis in susceptible persons. The committee noted that in many cases and for many conditions, evidence is still insufficient to conclude that such an association exists (IOM Committee on Damp Indoor Spaces and Health 2004).

The clinical effects of human exposure to mold spores were studied in sensitive subjects who had previously experienced potentially building-related symptoms at work. A highly controlled dose of fungal material was aerosolized directly from wet building materials. In a double-blinded study, eight sensitive school employees were exposed to *Penicillium chrysogenum* or *Trichoderma harzianum* spores for 6 min on 3 separate days. A statistically significant rise in symptoms from mucous membranes was assessed. This short-term exposure to high concentrations of two different molds induced no more reactions than exposure to placebo. Long-term experimental exposure studies on larger number of subjects would be needed to rule out an effect of mold exposure (Meyer et al. 2005).

One area in which the IOM panel felt evidence was insufficient to conclude whether an association or causal relationship concerned molds and a number of systemic conditions alleged to be related to mycotoxins (Fischer and Dott 2003). Molds can produce toxic metabolites known as mycotoxins. Over 400 mycotoxins have been described, most produced by species occurring on food. Many of the molds found indoors are similar to those on food and thus are also considered potential mycotoxin producers. It is important to note that mycotoxin production depends both on the growth conditions and the substrate, and therefore only a limited number of species are known to produce toxic compounds when grown on building or in house materials (Nielsen et al. 2002). The most well-known species is *Stachybotrys chartarum* but there has been considerable controversy regarding the toxic potential of *S. chartarum*. Care is essential when dealing with fungal problems caused by *Stachybotrys* or related fungi. Although the species *S. chartarum* is well known, there about 17 other different species of *Stachybotrys* and the related *Memnoniella* (Jarvis 2003; Jong and David 1976).

Research on the chemistry of *Stachybotrys* toxins is progressing to identify the chemical properties of species occurring in indoor environments. An excellent review of the toxins of *S. chartarum* describes a variety of secondary metabolites including trichothecenes, triprenylated phenols, and a new class of diterpenoids called “atranones” produced by the fungus (Jarvis 2003). Two chemotypes were found in *Stachybotrys*. The very toxic macrocyclic tri-chothecenes were detected in one-third of the isolates; less toxic, simple trichothecenes and a new class of atranones were found in the remaining two-thirds of the isolates. Atranones also possess significant biological activity (Miller J.D., personal communication). Species of *Chaetomium* and *Aspergillus vesicolor* are also potential toxin producers.

The clinical effects of mycotoxins have been alleged to include respiratory, neurologic, immunologic, dermatologic, gastrointestinal, and irritant effects, among others (Kuhn and Ghannoum 2003; Laumbach and Kipen 2005). Despite the absence of validated markers of exposure, efforts have been made to understand the relationship between mold exposures and chronic nonallergic health effects. There have also been trials of empiric therapies for treating mold-exposed individuals, including patients treated with cholestyramine (Shoemaker and House 2005). There remains a lack of consensus regarding the systemic effects of mold exposures (Terr 2004). One of the limiting factors in this research is reliable, validated markers of exposure to either molds or the putative mycotoxins.

In addition to intact molds and fungi, (1→3)-β-d-glucans are nonallergenic structural cell wall components of most fungi that have been suspected of playing a causal role in the development of respiratory symptoms associated with indoor fungal exposure. Current epidemiologic data do not permit conclusions to be drawn regarding the presence (or absence) of such an association between exposure and specific adverse health effects or which specific immunologic mechanisms underlie the presumed health effects (Douwes 2005).

Other biological hazards associated with indoor environments include bacteria, viruses, and other organisms. Although the association of *Legionella* with building water systems is well known, humidification systems carry risks for development of a variety of organisms capable of causing acute inflammatory responses as well as infection (Koschel et al. 2005). In addition, the design and operation of heating, ventilation, and air conditioning systems (HVACs) may have significant impact on the distribution of and subsequent exposures to aerosolized infectious agents (Li et al. 2005a, 2005b).

### Interactions and multiple exposures

Investigators have begun to measure multiple pollutants present within the same environment, including particles, combustion products, photochemical smog products, and allergens (Breysse et al. 2005; Hänninen et al. 2004a). This is partly because health effects are often related to multiple exposures and because many experimental interventions affect more than one exposure and agent. Important interactions also occur between exposures to pollutants and other hazards such as infectious agents. Exposures to O_3_ and NO_2_ have been shown to increase airway epithelial cell cytokine production (Spannhake et al. 2002). Studies have also demonstrated interactions between particles and other contaminants such as O_3_ that can potentiate the health effects of the two concomitant exposures (D’Amato et al. 2005; Harkema and Wagner 2005; Mar et al. 2005; Molhave et al. 2005). These findings suggest the possibility of additional benefits to interventions that reduce cumulative exposures to several pollutants compared with interventions focusing on only one exposure.

## Building Design and Health

There is growing interest in examining the interaction of building design and health (Cummins and Jackson 2001). Physical and design characteristics of built structures (lighting, heating, ergonomics, noise, design) may create additional exposures that might contribute to health and comfort. Some of these factors may also play a role in chronic health effects. For example, evidence indicates that suppression of melatonin by nocturnal artificial lighting may play a role in breast and colon cancer development (Pauley 2004; Stevens 2005).

Research in office buildings, which has tended to focus on health and productivity, is now moving beyond indoor air to issues such as office design and acoustics (De Croon et al. 2005). There is a growing literature on school design and injury prevention, with more recent research on physical activity, obesity, and the implications of school design for the development of chronic diseases in later life (Sallis and Glanz 2006), but there is limited literature on student achievement (Sexton et al. 2000). Finally, studies of residential building design have examined a range of health outcomes related to building design, notably injury, but also mental health and other outcomes (Bonnefoy et al. 2003; Weich et al. 2002).

## Intervention Studies

A number of investigators are now examining the effectiveness of environmental modification and education in reducing asthma severity. Examples include the use of air filters (Francis et al. 2003; Kilburn et al. 2003), pest management (McConnell et al. 2003), and education coupled with environmental modification (Krieger et al. 2002; Morgan et al. 2004; Tobias et al. 2004). Most of the interventions focus on control of more than one exposure, and have a relatively short duration.

Another study showed that use of ultraviolet germicidal irradiation within the HVAC system could reduce irritation symptoms in office workers (Menzies et al. 2003). This study was a crossover design in which subjects were blinded as to whether the intervention was in effect, and it used both symptom reporting and objective measures as outcomes. Although it did not examine all potential limitations and side effects of the intervention, it provides a useful example of the kinds of studies that may be needed to evaluate intervention strategies.

## Conclusion

It is increasingly apparent that indoor environments are unique and contain significant exposures that can affect the health of occupants. The exposures are the result of complex interactions between the structure, building systems, furnishings, the outdoor environment, and the building occupants and their activities. As people spend more time indoors, the opportunities increase for significant health effects resulting from these exposures. So too does the need for research into the circumstances that make exposures more likely and the effectiveness of interventions to reduce the exposures. Interventions may involve difficult tradeoffs such as increased ventilation versus the need for energy efficiency. In addition, more research is needed on the interactions of multiple exposures, and the risks to certain populations (such as children, the elderly, or socioeconomically disadvantaged populations). Identification of research priorities should include input from building designers, operators, and the public health community. Research on interventions should examine a range of outcomes and potential tradeoffs and confounders, and does not necessarily need to await the identification of specific causal agents. Research is also needed on better measures of dose, particularly for biological agents.
